# Cardiac Arrest Due to Kounis Syndrome Following Cephazolin Administration During Surgery Under Local Anesthesia

**DOI:** 10.7759/cureus.46297

**Published:** 2023-09-30

**Authors:** Ayaka Obata, Natsuko Saito-Sasaki, Hitomi Sugino, Misa Itamura, Etsuko Okada, Yu Sawada

**Affiliations:** 1 Dermatology, University of Occupational and Environmental Health, Kitakyushu, JPN

**Keywords:** literature review of diseases, clinical case report, cardiac arrest, cephazolin, kouns syndrome

## Abstract

The Kounis syndrome is described as the co-occurrence of allergic responses brought on by mast cell activation and acute coronary syndromes. We present a case of Kounis syndrome leading to cardiac arrest following the cephazolin sodium administration during the surgical resection of basal cell carcinoma. An 87-year-old woman was diagnosed with basal cell cancer. She received surgical excision of the tumor while anesthetized with lidocaine hydrochloride and 1% epinephrine. This patient began to itch around five minutes after^ ^cefazolin (CEZ) administration and eventually experienced cardiac arrest following diffuse rashes that spread throughout her body and edema in her eyelids. In line with the response, the electrocardiogram (ECG) also showed an elevated ST segment in V1-6, leading to possibly the diagnosis of Kounis syndrome. We also review the literature on Kounis syndrome following CEZ administration.

## Introduction

The degranulation of activated mast cells results in the release of various mediators, such as histamine [1], causing allergic responses. Kounis syndrome is described as the co-occurrence of allergic responses brought on by mast cell activation and acute coronary syndromes (ACS), such as vasospastic angina (VSA) and acute myocardial infarction [1-3]; however, this specific disease entity is not widely recognized as a critical event. Herein, we present a case of Kounis syndrome leading to cardiac arrest following cephazolin sodium (CEZ) administration during the surgical resection of basal cell carcinoma. Additionally, the detailed characteristics remain unclear, while several cases of Kounis syndrome following CEZ administration have been reported [4-6]. CEZ was especially administrated during the surgical resection, giving us a chance to evaluate ST-T segment change by a monitored electrocardiogram (ECG). Therefore, the ST-T segment change profiles in ECG might be a clue to identify a possible diagnosis of Kounis syndrome, as this etiology is uncommonly observed in clinical scenarios, especially during surgical operations. To clarify the detailed characteristics, we conducted a literature review of cases of Kounis syndrome after CEZ treatment to gain a better understanding of Kounis syndrome following CEZ administration.

## Case presentation

**Figure 1:**
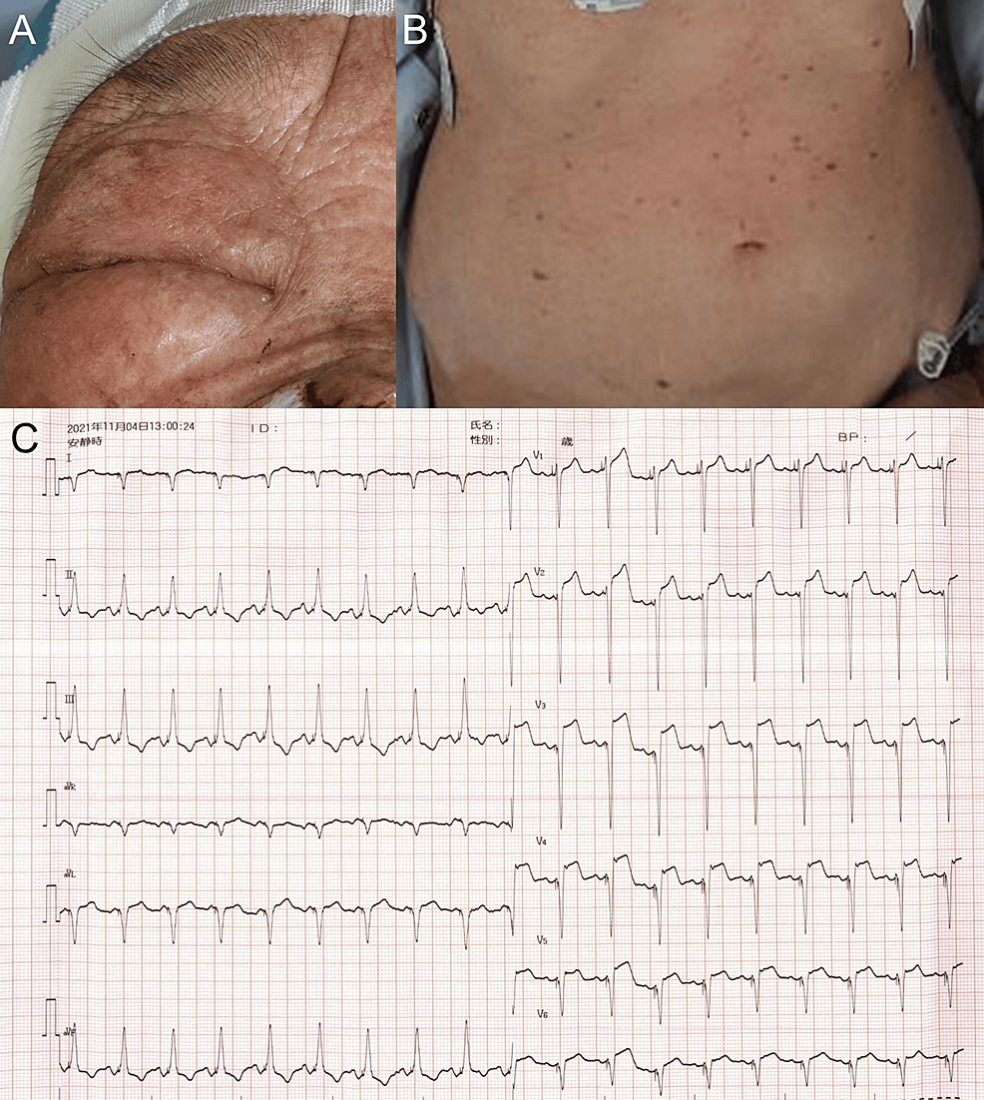
Clinical manifestations and ECG findings (A, B) Clinical manifestations. (A) Periorbital skin caused edema, and (B) diffused itch erythema was observed on the whole body. (C) An ECG analysis. ECG displayed an elevated ST segment in V1-V6.

An 87-year-old female was referred to our department for a hemorrhagic, black-based tumor with ulceration on the right nose wing, histologically diagnosed as basal cell carcinoma. She had a history of persistent ventricular tachycardia at age 77 and a myocardial infarction at age 63. Her nasal tumor required surgical resection, performed under local anesthetic lidocaine hydrochloride with 1% epinephrine. There was no skin wheal after the local anesthetic injection. However, the patient began to itch around five minutes after CEZ administration. Anesthesiologists were then called for a general state assessment of whether the patient might have initially experienced anaphylaxis. Despite the assistance of anesthesiologists and intensive care unit doctors, she had hypotension and chest pain, eventually experiencing cardiac arrest following diffuse rashes that spread throughout her body, edema in her eyelids, and a decreased oxygen saturation of the peripheral artery level that did not respond to oxygen administration (Figure 1A, 1B). In line with the response, the ECG also showed an elevated ST segment in V1-6 (Figure 1C). A biochemical blood test showed that a high-sensitivity troponin was slightly elevated at 0.390 ng/ml (normal range: 0.014). BUN levels were significantly elevated at 49 mg/ml (normal range: 8-20 mg/dl), as were creatinine levels at 1.79 mg/ml (normal range: 0.46-0.79 mg/dl), and LDH levels at 282 U/L (normal range: 124-222 U/L). According to echocardiography at the time, the left ventricular wall motion from the septum to the anterior wall reduced the dynamic movement with a low left ventricular ejection fraction.

Her symptoms recovered with continued infusions of adrenaline (0.5 mg/hr), hydrocortisone (200 mg), and methylprednisolone (125 mg) in addition to nicorandil drop infusion without any recurrence of symptoms. The ST segment also recovered without additional treatment.

Her ECG result naturally restored without further medical intervention. Furthermore, cardiologists in our hospital decided not to perform coronary arteriography (CAG) by cardiac catheter examination as her symptoms were relieved, and ECG and echocardiography findings improved to the level seen in the preoperative examination. This is despite the fact that prior case studies did not report a cardiac catheter examination as an essential examination to conduct to make a diagnosis of Kounis syndrome [7]. Although we strongly recommend conducting a CAG examination to make a diagnosis of Kounis syndrome and determine the subtypes, we could not obtain consent to conduct CAG from this patient. This patient declined to perform the CEZ skin test. A careful observation was conducted after the onset, she had no recurrence of the symptoms for 1 year as a possible diagnosis of Kounis syndrome.

## Discussion

Kounis syndrome is triggered by various allergic response causes [7-11]. Contrast agents (24%) and antibiotics (24%) are the most common causal agents, followed by muscle relaxant medications (14%). A recently updated study also showed a high prevalence of antibiotic use (48.0%) in the onset of Kounis syndrome [3]. Likewise, in our case, there are three cases of Kounis syndrome following CEZ administration including our case (Table 1) [4-6]. All these cases showed various coronary artery branches becoming the target for the cause of Kounis syndrome due to CEZ, indicating that we should keep in mind that various coronary artery branches become a target to cause Kounis syndrome in patients treated with CEZ. Among these cases, our case only had a history of cardiac disease and experienced cardiac arrest following CEZ administration.

**Table 1:** The summary of cases of Kounis syndrome following CEZ administration

Authors	Age/Sex	Past history of cardiac disease	Surgery	Outcome	ECG findings
Sequeira et al. [4]	56/Male	None	Knee arthroscopy for meniscus tear	Improved	Coronary artery spasm ST-segment elevation (II)
Adachi et al. [5]	92/Female	None	Bladder cancer surgery	Improved	Coronary artery spasm ST-segment elevation (II, III, and aVF)
Sato et al. [6]	69/Female	None	Bladder tumor	Improved	Coronary artery spasm ST depression (I, II, aVF, and V3–6)
Our case	87/Female	Ventricular tachycardia myocardial infarction	Basal cell carcinoma	Improved	Coronary artery spasm/cardiac arrest ST-segment elevation (V1-6)

## Conclusions

Therefore, clinicians should be informed that a past history of cardiac disease might be a risk factor for the onset of cardiac arrest in the course of Kounis syndrome during the perioperative use of CEZ. Although Kounis syndrome might be sometimes observed in clinical scenarios, dermatologists are not familiar with this phenomenon and should keep in mind the disease entity during surgical operations using antibiotic agents.
